# Characterization of Multifloral Bee Pollen Collected from Geographically and Botanically Distinct Regions in Tunisia

**DOI:** 10.3390/foods14233986

**Published:** 2025-11-21

**Authors:** Asma Sakhraoui, Fatma Arrari, Anis Sakhraoui, Volkan Aylanc, Maria Shantal Rodriguez-Flores, Maria Carmen Seijo, Miguel Vilas-Boas, Mondher Mejri, Soraia I. Falcão

**Affiliations:** 1 Laboratory of Functional Physiology and Valorization of Bio-Resources (LR23ES08), Higher Institute of Biotechnology, University of Jendouba, P.O. Box 382, Cedex Beja 9000, Tunisia; fatma.arrari90@gmail.com (F.A.);; 2 Department of Agricultural Production, Laboratory of Agricultural Production Systems and Sustainable Development (SPADD) (LR03AGR02), Higher School of Agriculture of Mograne, Carthage University, Moghrane 1121, Tunisia; anisak@alum.us.es; 3 Departamento de Biología Vegetal y Ecología, Universidad de Sevilla, Apartado 1095, 41080 Sevilla, Spain; 4 CIMO, LA SusTEC, Instituto Politécnico de Bragança, Campus de Santa Apolónia, 5300-253 Bragança, Portugal; volkan@ipb.pt (V.A.); mvboas@ipb.pt (M.V.-B.); 5 Departamento de Química e Bioquímica, Faculdade de Ciências, LAQV-REQUIMTE, Universidade do Porto, 4169-007 Porto, Portugal; 6 Department of Plant Biology and Soil Sciences, Universidade de Vigo, 32004 Ourense, Spain; mariasharodriguez@uvigo.gal (M.S.R.-F.); mcoello@uvigo.es (M.C.S.)

**Keywords:** bee pollen, nutritional composition, amino acids, fatty acids, minerals, total phenolics, Tunisia, protein content

## Abstract

Bee pollen is highly regarded for its nutritional and therapeutic properties, and Tunisia’s diverse ecosystems provide ideal conditions to produce high-quality bee pollen. The aim of this study was to characterize seven polyfloral bee pollen samples from major Tunisian regions, analysing their physicochemical and phytochemical parameters to evaluate compliance with national quality standards and their potential contribution to human nutrition. The nutritional and biochemical characterization of bee pollen samples was performed using standardized methods. Phenolic, flavonoid, and tannin contents were measured by colorimetric assays; carotenoids and chlorophylls spectrophotometrically; amino acids and sugars by HPLC; fatty acids by GC–MS; and minerals by atomic absorption spectroscopy. Amino acid levels were relatively constant between samples, but significant differences (*p* < 0.05) were noted, with concentrations ranging from 4.93 ± 0.15 mg·kg^−1^ (K-O4) to 82.72 ± 2.36 mg·kg^−1^ (O-O4). Tyrosine, aspartic acid, and glutamic acid were the dominant amino acids in both total and free forms, while threonine was identified as the relatively limiting amino acid. The proportion of total essential amino acids (TEAA) to total amino acids (TAA) met the nutritional recommendations set by the FAO. A total of 16 fatty acids were quantified in the seven BP samples, including nine saturated and six unsaturated fatty acids, with total content ranging from 0.26 g/100 g^−1^ (T-03) to 37.06 g/100 g^−1^ (G-03), which the primary fatty acids identified were α-linolenic acid, palmitic acid, and oleic acid. However, palmitoleic acid was detected in only two samples, in small amounts (0.34% and 0.46%). Essential minerals such as K, Ca, P, Mg, Zn, Fe, Mn, and Cu were present in significant amounts, playing a crucial role in both plant metabolism and human health Despite variations between samples, Tunisian bee pollen was overall evaluated as a valuable dietary supplement.

## 1. Introduction

Pollen is often regarded as one of the most nutritionally valuable natural products and is frequently referred to as a “complete food” due to its rich nutritional and therapeutic properties [1]. Pollen is a natural product collected by bees from flowers. Once gathered and transported back to the hive in the form of compact granules, it is referred to as bee pollen (BP) [2]. Bees collect pollen grains from flowers and compact them with nectar and salivary secretions; once transported back to the hive in the form of granules, this product is known as bee pollen (BP) [3]. Bee pollen is therefore the harvested form of floral pollen gathered and processed by bees. Its composition and visual characteristics vary significantly depending on the botanical origin, as pollen grains from different plant species differ in shape, colour, size, and weight contributing to the high variability observed in bee pollent [4]. The chemical compositions of bee pollen have drawn worldwide research interest, covering broad aspects ranging from plant physiology to biochemistry [5]. More than 200 compounds have been found in the bee pollen of various botanical origins, including proteins, essential amino acids, carbohydrates, lipids and fatty acids, phenolic compounds, enzymes and coenzymes, and vitamins [6,7] and small amount of volatiles which can act as strong antioxidants [8,9]. Numerous studies indicate that the antioxidant activity of bee products is variable and generally depends on the type and source of flowers, geographical origin, climatic conditions, processing, and storage [10,11]. Pollen consists of male reproductive cells of the seed plants, formed in flower thecae [12]. The nutritional value of pollen is often evaluated by the protein concentration (10–40% in dry weight), carbohydrates (13–55% in dry weight), lipids (1–13% in dry weight), dietary fibers (0.3–20% in dry weight), phenolic compounds (up to 2.5% in dry weight) [13], as well as the presence and quantity of essential amino acids 10.4% [14,15]. Some pollen types are classified as highly nutritious, while others exhibit a marginal value [16].

In Tunisia, beekeeping is a well-established agricultural activity spanning from Mediterranean coastal ecosystems to the arid southern regions, supported by the country’s rich and varied flora [17,18]. This ecological diversity provides favourable conditions for the production of bee pollen with distinctive botanical origins and chemical profiles [19]. Tunisia is considered among the leading bee pollen producers in the Mediterranean basin and Africa, with approximately 13,000 beekeepers managing around 305,000 hives, contributing about 0.1% of the national GDP and 1% of the agricultural GDP [20,21]. However, despite this potential, scientific investigations into the detailed nutritional composition of Tunisian bee pollen remain limited. Most previous studies have focused on ethnopharmacological surveys or melissopalynological/physicochemical characterizations of bee pollen in Tunisia [19]. By contrast, comprehensive biochemical analyses covering proteins, lipids, carbohydrates, amino acids, fatty acids, sugars, phenolics, and mineral elements are still scarce. Understanding the nutritional quality of Tunisian bee pollen is of particular interest for several reasons. Therefore, the present study aimed to characterize the biochemical and nutritional composition of seven multifloral bee pollen varieties collected from major production regions across Tunisia. The analyses included the determination of proximate composition (proteins, lipids, carbohydrates, and dietary fiber), total phenolic content, amino acids, fatty acid profiles, mineral elements, and bioactive compounds across seven geographically distinct Tunisian regions. This study introduces novel insights into regional variation and expands current knowledge on the nutritional diversity of Tunisian bee pollen. By comparing samples from different regions, this study sought to highlight both the compositional variability and the overall nutritional value of Tunisian bee pollen. The findings provide valuable insights for the scientific characterization and potential commercial valorisation of Tunisian bee pollen as a high-quality natural product.

## 2. Materials and Methods

### 2.1. Chemicals and Reagents

Methanol, sulphuric acid, petroleum ether, toluene, pentanoic acid, diethyl ether, acetonitrile, potassium phosphate, sodium bicarbonate, sodium hydroxide, hydrochloric acid, and calcium chloride dihydrate were purchased from Fisher Scientific (Pittsburgh, PA, USA). Kjeldahl catalyst tablets, potassium chloride, sodium chloride, and ammonium carbonate were purchased from Panreac Applichem (Barcelona, Spain). Magnesium chloride hexahydrate was purchased from Acros Organics (Pittsburgh, PA, USA).

### 2.2. Bee Pollen Samples

**Figure 1:**
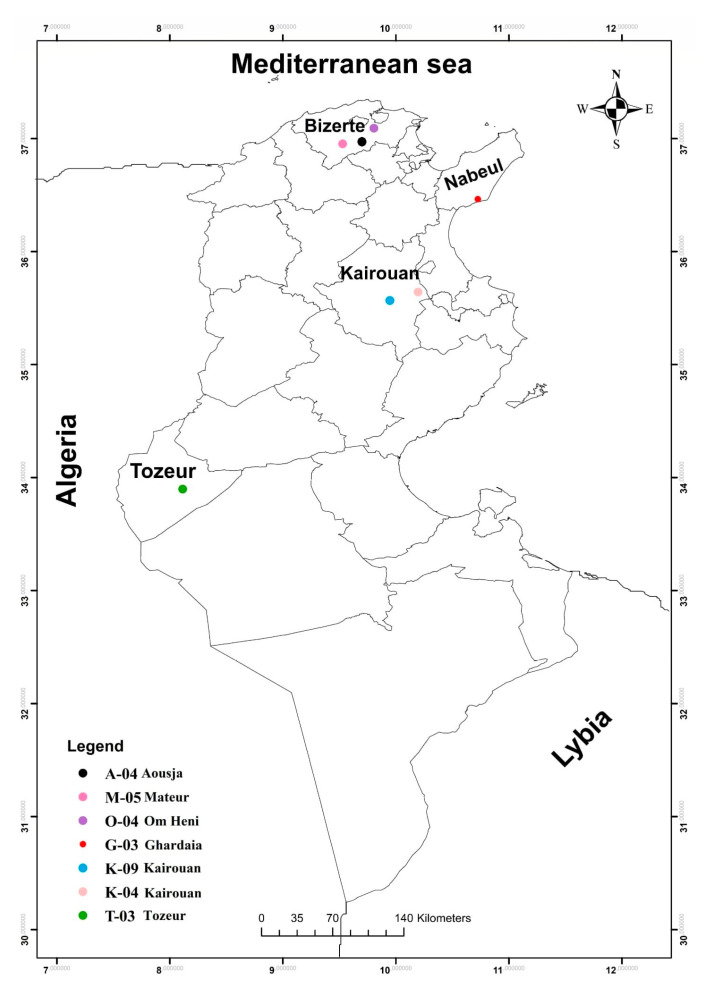
Collection sites of bee pollen from Tunisia.

Three BP samples were collected from hives located at seven different apiaries representing distinct bioclimatic zones in Tunisia (Figure 1). The map of bee pollen (BP) collection sites in Tunisia was created using ArcGIS software version 10.6.1 (Esri^®^, Redlands, CA, USA). The collection sites included Bizerte-Aousja (A-04), Nabeul-Ghardiai (G-03), Kairouan (K-04), Kairouan-Weslatia (K-09), Bizerte-Mateur (M-05), Bizerte-Om Heni (O-04), and Tozeur (T-03). These regions differ markedly in climatic and environmental conditions, ranging from humid coastal zones in the North (Bizerte, Nabeul) to semi-arid and arid zones in the Centre and South (Kairouan, Tozeur). The geographical coordinates (latitude, longitude, altitude) and specific characteristics of each sampling site are provided in Supplementary Table S1. Samples were collected by local beekeepers following hygienic procedures according to HACCP guidelines and received as fresh pollen pellets. No drying was performed to preserve heat- and air-sensitive nutrients and bioactive compounds, ensuring that the biochemical and nutritional analyses reflect the natural composition of the pollen. Each sample was carefully cleaned to remove debris (wood particles and bee parts), then stored in airtight glass containers at 4 °C and analysed within two weeks of collection. For subsequent analyses, the samples were ground to a fine powder (20 mesh) using a Moulinex A320 blender (Groupe SEB, Mayenne, France).

### 2.3. Palynological Analysis

The botanical origin of the bee pollen samples was determined using the Standard Methods for Pollen Research proposed by Campos et al. [22]. Each homogenised sample (1 g) was divided into subsamples according to its distinct colours, reflecting different floral sources. The colour-based subsamples were weighed, suspended in 10 mL of distilled water and shaken for 10 min. They were then centrifuged at 4500 rpm for a further 10 min. An aliquot of 100 μL of the sediment was used to prepare microscope slides. The botanical origin of each subsample was identified by comparison with a reference collection of pollen slides and standard identification keys using an optical microscope (Nikon Optiphot II, UK Ltd., London, UK). This colour-based microscopic method follows the recommendations of Campos et al. [22], which emphasise the use of morphological identification supported by reference collections to ensure accuracy and reproducibility in determining the floral origin of bee pollen [23]. The pollen spectrum of each sample was determined by considering the relative weight of each colour-based subsample and its botanical composition. The total number of pollen types and their corresponding botanical families were recorded for each sample, and the dominant pollen types were used to characterise the floral origin of the bee pollen. The results were expressed as percentages.

### 2.4. Preparation of Aqueous Extract of Bee Pollen (AEBP)

To prepare the aqueous extract of bee pollen, 5 g of BP were combined with 50 mL of distilled water. This mixture was incubated at 25 °C (room temperature) in a temperature-controlled incubator (Memmert INB 200, Schwabach, Germany) for 24 h with continuous magnetic stirring at 300 rpm using a magnetic stirrer (IKA RCT Basic, Staufen, Germany). Following incubation, the mixture was centrifuged at 10,000× *g* for 10 min using a Sigma 3-18KS centrifuge, Osterode am Harz, Germany, and the supernatant was collected. The resulting solution was then filtered through Whatman No. 1 filter paper to remove any remaining particulate matter. The clarified extract was lyophilized using a Refrigerated Vapor Trap (RVT450, Labconco, Kansas City, MO, USA). Finally, the lyophilized extract was carefully weighed and portioned into dry aliquots in pre-labelled microcentrifuge tubes, which were then stored in a dark environment at 4 °C until further use.

### 2.5. Free Acidity and pH

To measure the pH, 2 g of bee pollen were mixed with 5 mL of distilled water and thoroughly homogenized using a vortex mixer for 1–2 min to ensure uniform dispersion. The pH of the resulting suspension was measured using a pH meter (Hanna Instruments, HI5521-02 Model, Smithfield, RI, USA). The free acidity of the samples was achieved by titration of the homogenized suspension to pH 8.30 with 0.05 mol. mL^−1^ NaOH using a potentiometric titrator (Hanna Instruments, HI 902 Model, Smithfield, RI, USA).

### 2.6. Nutritional, Chemical Composition and Energy Value

The nutritional composition of bee pollen, including water, ash, lipid, protein, and dietary fiber, was determined according to the procedures proposed by the Association of Official Analytical Chemists (AOAC, 2016) [24]. The water content was determined using a moisture analyser (PMB 53 Moisture Analyzer, Adam Equipment, Milton Keynes, UK) following the AOAC 925.45 Method. Ash content was determined by incineration (Ivymen Furnace N-22L, JP Selecta, Barcelona, Spain) at 550 ± 5 °C a muffle furnace (Ivymen Furnace N-22L, JP Selecta, Barcelona, Spain) according to the AOAC 923.05 Method. The residue remaining after combustion was weighed to calculate the ash content. Total lipid content was extracted using a Soxhlet apparatus with petroleum ether as the solvent, following AOAC 989.05. The extracted lipid was dried and weighed to determine the lipid percentage.The protein content was estimated using the macro-Kjeldahl technique (N × 6.25, for both bee products or blank) with an automatic Kjeldahl steam distillation unit (Pro-Nitro A, JP Selecta, Barcelona, Spain), following the AOAC 920.87 Method. Both raw and digested samples were analysed. The estimated (by difference) available carbohydrate (FAO/WHO, 1998) and total energy value [25] of BP were calculated using the following Equations (1) and (2), respectively:

(1) Total available carbohydrates (%) = 100 − (g ashes + g proteins + g lipids + dietary fiber)

Energy value (kcal/100 g) was calculated using the Atwater coefficients [25]:

(2) *Energy* (kcal) = 4 × (g protein + g carbohydrate) + 2 × (g dietary fiber) + 9 × (g lipids)

### 2.7. Phytochemical Analysis of Secondary Metabolites

Phytochemical analysis was conducted following the protocols outlined by Trease and Evans [26]. The tests were based on precipitation or colour reactions and were carried out as follows:

**Tannins**: A few drops of FeCl_3_ (1%) are added to 2 mL of the aqueous bee pollen extract. The formation of a green precipitate indicates the presence of tannins.

**Phlobotannins**: A mixture of 2 mL of aqueous bee pollen extract and 2 mL of HCl (1%) was heated in a water bath at 100 °C for 5 min. The appearance of a red precipitate confirms the presence of phlobotannins. **Steroids**: A solution of 2 mL of aqueous extract and 2 mL of chloroform is prepared, followed by the addition of 2 mL of sulfuric acid. A red coloration in the chloroform phase indicates the presence of steroids.

**Terpenoids:** 2 mL of the extract were mixed with 2 mL of chloroform, heated at 100 °C until complete evaporation, and then 2 mL of concentrated sulfuric acid were added. The development of a grey coloration indicated the presence of terpenoids.

**Alkaloids:** 1 mL of the extract was treated with five drops of Wagner’s reagent. The formation of a brown precipitate confirmed the presence of alkaloids.

**Saponins**: To assess the presence of saponins, 10 mL of bee pollen aqueous extract are vigorously shaken for 15 min, and the foam height is measured after 15 min of rest:

A foam height of less than 5 mm indicates the absence of saponins.

A height between 5 and 9 mm suggests a low concentration of saponins.

### 2.8. Determination of Total Phenolics, Flavonoids, and Condensed Tannins

#### 2.8.1. Total Phenolic Content

Total phenolic compounds present in the hydroethanolic extracts were quantified spectrophotometrically through the Folin–Ciocalteu test following the protocol of Singleton [27] with some modifications. Briefly, 0.5 mL of the AEBP was mixed with 10% (*v*/*v* Folin–Ciocalteu’s reagent (0.25 mL). After 3 min, 1 mL of 7.5% (*w*/*v*) sodium carbonate (Na_2_CO_3_) solution was added to the mixture, and the volume was made up to 5 mL with distilled water. The solution was kept for 10 min at 70 °C to prevent degradation of phenolic compounds, and then cooled in the dark for 20 min. Thereafter, the mixture was centrifuged for 10 min at 5000 rpm and only the supernatant was used for the assay. Absorbance was measured at 760 nm using a UV–Vis spectrophotometer (Shimadzu UV-1800, Kyoto, Japan). A solution of methanol was used as the blank, and gallic acid was used as the standard (0.001–0.25 mg·mL^−1^) for constructing the calibration curve (y = 8.1477x + 0.0205; R^2^ = 0.9998). The total phenolic content (TPC) was expressed as mg of Gallic acid equivalents (GAE) per g of bee pollen. The analysis was performed in triplicate [28].

#### 2.8.2. Total Flavonoid Content

The aluminium chloride method, previously described by Zhishen et al. [29], was used to determine the flavonoid content of the samples. Briefly, 0.2 mL of sample (5 mg·mL^−1^) was mixed with 0.2 mL of AlCl_3_ solution (2% aluminium chloride in 5% acetic acid/methanol). After that, 2.8 mL of methanol with 5% acetic glacial acid was added. The mixture was then incubated for 30 min in the dark, and the absorbance was measured at 415 nm. A mixture of sample and methanol (5% acetic glacial acid) was used as the blank sample. Standard solutions of quercetin (0.0016–0.05 mg∙mL^−1^) were used for constructing the calibration curve (y = 4.4625x + 0.0031; R^2^ = 0.9992). The total flavonoid content (TFC) was expressed as mg of quercetin equivalents (QE) per g of bee pollen. The experiment was run in triplicate.

#### 2.8.3. Determination of Condensed Tannins

The condensed tannin content of the aqueous bee pollen extract (AEBP) was determined using the vanillin-HCl colorimetric assay [28]. This method is based on vanillin’s ability to react with condensed tannin molecules in the presence of acid, forming a coloured complex that is measured at 550 nm. Briefly, 1 mL of bee pollen extract was mixed with 5 mL of vanillin reagent and 1 mL of concentrated HCl. The mixture was incubated at 25 °C in the dark for 20 min, allowing vanillin to react with condensed tannins to form a red-coloured complex. The absorbance was measured at 550 nm using a UV–Vis spectrophotometer (Shimadzu UV-1800, Kyoto, Japan). A calibration curve was established using catechin as a standard to quantify the condensed tannins. A mixture of sample and methanol was used as the blank. The concentration of condensed tannins in BPAE is expressed as milligrams of catechin equivalent per gram of dry matter (mg CE·g^−1^ DW).

### 2.9. Total Fiber

The fiber content of BP was determined using the Weende method [30]. Specifically, 2.5 g of powdered sample was boiled in 100 mL of formic acid (80% *v*/*v*) for 75 min. The mixture was then cooled and filtered using a Büchner funnel. The insoluble fraction was collected, and the resulting residue was dried in an oven at 103 °C for 3 h before being incinerated in a muffle furnace at 550 °C for another 3 h. After cooling, the extracts were weighed to determine the ash mass.

### 2.10. Quantification of Carotenoids and Chlorophyll

BP solutions (5 mg·mL^−1^) were prepared by dispersing the powdered sample in 70% methanol, followed by sonicating for 15 min to facilitate pigment extraction. The mixture was then centrifuged at 5000 rpm for 10 min (Eppendorf 5810R, Hamburg, Germany) to separate insoluble material. The resulting supernatant was collected and filtered through Whatman No. 1 filter paper to remove remaining particulates. The clear extract was analyzed in triplicate. Carotenoid content was quantified by measuring absorbance at 470 nm, while chlorophyll a and b concentrations were determined at 653 nm and 666 nm, respectively, using a UV-Vis spectrophotometer (Shimadzu UV-1800, Kyoto, Japan) [31]. The concentrations of total carotenoids and chlorophyll a and b were calculated using the equations proposed by Lichtenthaler and Wellburn [31].

### 2.11. Fatty Acids Profile

The samples were extracted with petroleum ether in a Soxhlet apparatus for 4 h. Fatty acids were determined by gas–liquid chromatography with mass spectrometry detection (GC–MS) based on the following trans-esterification procedure: fatty acids were methylated with 4.45 mL of methanol: sulphuricacid: toluene 2:1:1 (*v*:*v*:*v*) and 0.55 mL of internal standard (pentanoic acid; 0.5 mg·mL^−1^), for 12 h in a water bath at 50 °C and 160 rpm; then, 3 mL of deionized water was added, to obtain phase separation; the fatty acid methyl esters were recovered with 3 mL of diethyl ether by shaking in a vortex, and the upper phase was passed through a micro-column of sodium sulphate anhydrous to eliminate the water; the sample was recovered in a vial, and filtered before injection with 0.22 µm nylon filter.

The fatty acid profile was analysed with a Perkin Elmer system (GC Clarus^®^ 580 GC module and Clarus^®^ SQ 8 S MS module, Shelton, CT, USA) gas chromatograph, equipped with DB-WAX fused-silica column (30 m × 0.25 mm i.d., film thickness 0.25 μm; J & W Scientific, Inc., Folsom, CA, USA), and interfaced with a Perkin-Elmer Turbomass mass spectrometer (software version 6.1, Perkin Elmer, Shelton, CT, USA). The oven temperature was programmed as 50 °C for 1 min, 50–200 °C, at 25 °C·min^−1^, and subsequently at 3 °C·min^−1^ up to 230 °C, and then held isothermal for an additional 23 min. The transfer line temperature was set as 250 °C; ion source temperature, 230 °C; carrier gas, helium, adjusted to a linear velocity of 1 mL·min^−1^; ionization energy, 70 eV; scan range, 40–300 u; scan time, 1 s. Split injection (1:50) was carried out at 250 °C. For each analysis, 1 µL of the sample was injected in the GC. The peaks identification was based either on: (i) the comparison of the obtained spectra with those of the NIST mass spectral library; (ii) confirmed using the linear retention indices calculated from the retention times of an n-alkane mixture (C7–C40) (Supelco, Bellefonte, PA, USA) analysed under identical conditions; (iii) with the comparison with published data, and when possible; (iv) with commercial standards. The quantitation was carried out using the relative values directly obtained from peak total ion current (TIC) peak area relative to the internal standard. The results were expressed as g per 100 g of sample DW.

### 2.12. Amino Acids Profile

Amino acids were quantified following acid hydrolysis and HPLC-FLD analysis. Briefly, 0.5 g of bee pollen powder was mixed with 4 mL of HCl (6N) in sealed tubes and hydrolyzed at 105 °C for 24 h. After cooling, samples were neutralized with 6 mL of sodium hydroxide (6N). The hydrolysates were first filtered through Whatman No. 1 filter paper, followed by a 0.22 µm syringe membrane filter, and transferred to HPLC vials.

A mixed amino acid standard containing 19 amino acids (alanine, valine, leucine, isoleucine, threonine, tryptophan, methionine, phenylalanine, tyrosine, serine, aspartic acid, glutamic acid, asparagine, glutamine, lysine, arginine, histidine, glycine, and proline) was prepared for quantification.

The amino acid composition of bee pollen was analysed using high-performance liquid chromatography with fluorescence detection (HPLC-FLD) through pre-column derivatization with ortho-phthalaldehyde (OPA, 10 mg·mL^−1^) prior to injection. Amino acid separation and quantification were performed on an Agilent 1200 liquid chromatography system. The chromatographic analysis was conducted using a C18 column (250 × 4.6 mm; 5 µm bead size) at a temperature of 43 °C, with a fluorescence detector (λEX = 340 nm, λEM = 440 nm), a flow rate of 1 mL·min^−1^, mobile phase A (ACN-MeOH-H_2_O, 45/45/10, *v*/*v*/*v*), and mobile phase B (Na_2_HPO_4_, 2.75 g·L^−1^, pH 6.5).

### 2.13. Mineral Content Analysis

The mineral elements were analysed by atomic absorption spectroscopy (AAS) using a Perkin Elmer PinAAcle 900 T Spectrometer (Waltham, MA, USA). Potassium, sodium, calcium, magnesium, zinc, and iron were analysed by flame ionization AAS, while atomic absorption spectrophotometry in a graphite chamber was applied for manganese, copper, cadmium, and lead. The sample preparation was carried out through microwave-assisted extraction, using a MARS 5 Digestion Microwave System (CEM Corporation, Matthews, NC, USA). Approximately 1 g of the sample was weighed into a PTFE digestion tube followed by the addition of 10 mL of concentrated nitric acid. The digestion was performed by setting the ramp temperature program: 15 min until 200 °C with a power of 1200 W, followed by additional 15 min at the same temperature and power conditions. After cooling down, the resulting solutions were diluted up to 50 mL with deionized water and analysed by AAS, with prior treatment for specific elements. For the determination of potassium and sodium, the sample was diluted in a caesium chloride solution (1 g·L^−1^); for calcium and magnesium, the sample was diluted in a lanthanum chloride solution (1 g·L^−1^); for manganese and copper, a magnesium nitrate solution (1 g·L^−1^) was used as a matrix modifier; and iron and zinc were directly analysed. The quantification of elements was achieved by comparing the absorbance responses with a calibration prepared from commercial standard solutions (Panreac Applichem, Barcelona, Spain) within the following ranges: Ca, Fe and K (0.25 to 5 ppm), Na (0.125 to 2.5 ppm) Mg and Zn (0.0625 to 1.25 ppm), Cu and Pb (25 to 100 ppb), Mn (10 to 40 ppb) and Cd (1.25 to 4 ppb).

### 2.14. Data Analysis

Each assay was conducted in a completely randomized design (CRD) with three replications and one factor (pollen origin). Deviations from the mean was expressed as standard errors (SE). All statistical analyses were performed using IBM SPSS V. 25 (SPSS Inc. Chicago, IL, USA) for Windows, applying a significance level (α) of 0.05. The Kolmogorov–Smirnov test was performed to check for validity of the normality assumption and Levene’s test for the homogeneity of variance. To meet the assumption of homogeneity of variances for parametric tests, C8:0, C10:0, C14, C18:2 and C20:0 values were transformed using √x function. The main univariate differences were evaluated for each functional BP trait with general linear models (LMs). This approach extends the traditional ANOVA framework, allowing a flexible treatment of continuous and potentially heteroscedastic data [32]. Post-hoc pairwise comparisons were performed using the Bonferroni–Dunn test. When homogeneity of variance was not achieved after data transformation, univariate differences were analysed using the γ generalized linear model (GLM) with Wald’s χ^2^ [32]. Principal Components Analysis (PCA) was carried out using Past Software version 5.3 (Natural History Museum, Oslo, Norway) [33] analysing the correlation matrix with 25 maximum iterations for convergence without rotation to extract independent PCA factors with eigenvalues >1.

## 3. Results

### 3.1. Palynological Analysis

The palynological analysis, conducted using the methodology outlined by Campos et al. [22], enabled the botanical origin and pollen composition of the bee pollen (BP) samples to be identified. A total of 47 pollen types belonging to 25 botanical families were identified, confirming the multifloral nature of all the samples analysed, since none were dominated by a single pollen type.

The most frequent pollen types were the *Taraxacum* and *Anthemis* types (both Asteraceae), and the *Papaver* type (Papaveraceae), which together represented the main floral sources contributing to the overall composition of Tunisian bee pollen. Other recurrent pollen types included *Helianthus annuus*, *Artemisia*, *Carduus*, *Sinapis alba*, the *Brassica* type (Brassicaceae), *Cistus* (Cistaceae), *Hedysarum* (Fabaceae), *Olea europaea* (Oleaceae) and *Chenopodium* (Chenopodiaceae), reflecting the botanical richness typical of Mediterranean ecosystems.

**Figure 2:**
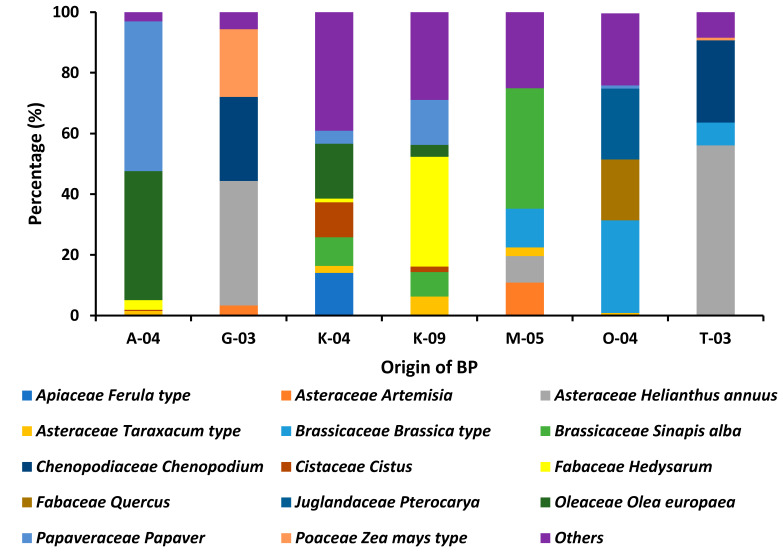
Palynological analysis: Plant species contributing predominant, secondary, significant minor, and minor pollen types in the analyzed BP samples from different regions of Tunisia Physicochemical Characterization of Bee Pollen.

Significant differences were observed between regions (Figure 2), illustrating the strong influence of local flora and bioclimatic gradients on pollen composition. Samples from the north of the country (Bizerte and Nabeul) contained mainly pollen from the families Apiaceae, Brassicaceae and Asteraceae, which are associated with temperate Mediterranean vegetation and cultivated crops. In contrast, central regions (e.g., Kairouan) exhibited a more balanced representation of Fabaceae, Cistaceae and Brassicaceae, reflecting transitional ecosystems combining shrublands and agricultural areas. In the southern arid zone (Tozeur), *Helianthus annuus* pollen predominated, consistent with the dominance of xerophytic and annual species adapted to dry environments.

These findings confirm the multifloral nature of Tunisian bee pollen and the botanical specificity associated with each bioclimatic region. This variability defines the geographical signature of the samples and explains the differences in their nutritional and antioxidant properties, as the chemical composition of bee pollen is closely tied to its floral origin. This compositional variability is crucial for understanding the nutritional and bioactive potential of Tunisian bee pollen, since different pollen sources contribute different phenolic, amino acid and lipid profiles [34,35]. Overall, the palynological data provide essential insights into the geographical and botanical origins of the samples, supporting their authentication and promoting the valorisation of Tunisian bee pollen as a nutritionally rich, multifunctional natural product.

### 3.2. Physicochemical Characterization of Bee Pollen

The physicochemical and nutritional analyses of the bee pollen samples demonstrate significant variability in protein, carbohydrate, lipid, and energy content across the different regions. First, it contributes to the valorisation of local apicultural products by providing scientific evidence of their health-promoting properties. Second, such knowledge can support quality standardization and labelling initiatives, which are crucial for domestic and export markets. Finally, it enriches our understanding of how environmental and botanical diversity influence the nutritional profiles of bee-derived products, offering valuable insights for both producers and consumers seeking natural and sustainable sources of nutrition.

The pH is a key factor influencing the stability and quality of bee pollen by affecting its susceptibility to microbial growth and degradation [36]. In this study, pH values ranged from 4.54 for T-03 to 5.46 for K-09 (Table 1), with a mean of 4.30 (*p* < 0.05). These results are consistent with those reported for bee pollen from Tuscany, Portugal, Greece, and India [34,37], confirming its naturally acidic character, which helps inhibit microbial development and preserve product stability. The acidity of BP is mainly attributed to the presence of organic acids such as oxalic, succinic, malic, citric, ptartaric, gluconic, and lactic acids found in these natural bee products [9,38]. The ash content is an important quality parameter that can vary depending on to the botanical origin of the pollen. In the present study, the ash content of the analyzed samples ranged between 1.89 ± 0.04% and 2.78 ± 0.07% (*p* < 0.05), with an average of 2.18%, which is in good agreement with previously reported data [39]. Among the samples, K-04 exhibited the lowest ash content (1.89 ± 0.04%), whereas M-05 recorded the highest value (2.78 ± 0.07%). Overall, the results for the ash content of BP samples in the current study agree with the data from previously reported works; the average ash content in the pollen is in the range from 2.27% to 3.5% [40]. Recommendations for BP refer that it should not exceed 6% [7]. The water content of BP is an important factor which may affect the activity of microorganisms, deterioration, and the shelf life of these products and so, for consumption, it is normally subject to previous dehydration. BP samples showed values that varied between 6.03 and 9.53 g·100 g^−1^ for K-04 and T-03, respectively. The water content for BP samples was higher than the commercially proposed maximum limit (6 g·100 g^−1^) [7]. In the case of pollen subjected to drying, the moisture content ranges from 2% to 9%. Commercial pollen contains 5.91% of water [1], while the data presented by Oroian et al. [39] showed 3.47% water content in the pollen coming from Romania.

Lipids are one of the main macronutrients with a significant contribution to the nutritional value of BP and vary depending on the botanical origin [41,42]. Lipid content was significantly affected by BP origins (*p* < 0.05). The highest lipid content in BP samples was found for M-05 with a value of 4.43 g·100 g^−1^ while the lowest lipid content was recorded in G-03 with a value of 1.60 ± 0.05 g·100 g^−1^. Our findings are in line with Li et al. [43] reporting that pollen lipids make up to 13 g·100 g^−1^, and their content and variety depend on the plant source. In addition, our findings were consistent with the results reported for BP in different countries [9,44].

The protein content of the bee pollen samples ranged from 15.74% to 27.92% (*p* < 0.05), with an average value of 22.31%, confirming the nutritional importance of bee pollen as a valuable source of dietary protein. This high protein content supports its classification as a nutrient-dense food, capable of contributing significantly to human and animal nutrition. The values obtained in this study are consistent with those reported in the literature for bee pollen from different regions, including Brazil (12.28–27.07%) [45], Spain (15.19–20.23%), Colombia (21.6%), and Italy (19.5%) [46]. However, Gardana et al. [46] observed a slightly lower protein concentration (12.3%) in Spanish bee pollen. Such variations in protein content are commonly attributed to differences in botanical and geographical origin, climatic conditions, harvest period, and storage parameters, all of which influence pollen composition and nutrient accumulation. Furthermore, the high protein levels observed highlight the potential of bee pollen as a functional ingredient in health-promoting diets.

Carbohydrates were the main macronutrient in all bee pollen samples, ranging from 43.4 g/100 g (A-04) to 62.7 g/100 g (T-03), providing a significant energy source. Variability among samples likely reflects differences in floral origin, climate, and harvesting period, consistent with previous reports from other regions [39,45].

The energy value of the bee pollen samples varied from 354.0 ± 8.3 to 373.9 ± 3.8 kcal/100 g (*p* < 0.05), with an average of 350.16 kcal/100 g, confirming that bee pollen is an energy-dense food. This caloric value mainly arises from its balanced composition of carbohydrates, proteins, and lipids, highlighting its role as a natural nutritional supplement capable of supporting metabolic activity and general well-being.

### 3.3. Phytochemical Characterization of Bee Pollen

The phytochemical analysis of different pollen origins (T-03, K-09, K-04, M-05, A-04, O-04, G-03) reveals the presence of tannins, phlobotannins, terpenoids, alkaloids, steroids, and saponins in varying intensities, while alkaloids and terpenoids are present in average amounts across all samples (+ to +++) (Table 2). Tannins, phlobotannins, terpenoids, and steroids are consistently present, suggesting antioxidant, astringent, and anti-inflammatory potential [47]. However, A-04 exhibits the highest amount (+++), whereas saponins show inconsistent distribution, possibly affecting immune modulation and cholesterol regulation [48]. Our results are consistent with those of Kaur et al. [49], who reported that the different extraction methods showed that water was the best solvent for extracting the most components from bee pollen, followed by ethanol and methanol. Furthermore, our results were in line with those of BP’s different origin reports that an area’s location and seasons have a significant impact on the chemistry of bee pollen. Each sample has a different range of these phytochemicals because of variations in the place and season [50]. These findings indicate that different pollen sources contribute variably to bioactive potential, warranting further biological and functional analyses to better assess their pharmacological applications.

### 3.4. Secondary Metabolites Quantities

#### Total Phenolic, Total Flavonoids and Condensed Tannin Compounds

The phytochemical compound content in pollen samples from different origins is detailed in Table 3, illustrating the concentrations of total phenolic compounds (mg GAE·g DW and total flavonoids (mg QE·g DW) in bee pollen samples. The TPC varied significantly (*p* < 0.05) between 4.54 ± 0.04 and 5.15 ± 0.08 mg GAE/g DW, while the TFC ranged from 3.27 ± 0.2 to 3.52 ± 0.002 mg QE/g DW. Phenolic compounds are significantly more abundant than flavonoids in all samples. This indicates that other phenolic compounds, beyond flavonoids, contribute substantially to the total polyphenol content [51]. The O-04 sample exhibited the lowest polyphenolic content (4.54 ± 0.04 mg GAE·g DW), indicating variability in its composition compared to sample K-09, which contained 5.46 ± 0.05 mg GAE·g DW. However, the total flavonoid content (TFC) showed relatively minor differences among the samples, ranging from 3.27 ± 0.20 mg QE/g DW in K-04 to 3.52 ± 0.002 mg QE/g DW in K-09. The other samples exhibited comparable values: G-03 (3.49 ± 0.002 mg QE/g DW), T-03 (3.50 ± 0.002 mg QE/g DW), O-04 (3.37 ± 0.001 mg QE/g DW), M-05 (3.51 ± 0.001 mg QE/g DW), and A-04 (3.44 ± 0.005 mg QE/g DW). These significant differences among pollen extracts from different sources likely reflect variations in their botanical origins and environmental or climatic conditions. The observed TPC values are comparable to those reported for Indian bee pollen (9.79–35.63 mg GAE/g) [36] and Brazilian pollen (6.50–29.20 mg GAE/g) [52]. Likewise, the TFC values align with previous reports for Brazilian bee pollen (0.30–17.50 mg QE/g) [52] and Indian bee pollen (9.72–15.62 mg QE/g) [36], supporting the influence of geographical and floral factors on the phenolic and flavonoid composition of bee pollen. These findings highlight the rich polyphenol composition of pollen, which may enhance its antioxidant properties and potential health benefits [53,54]. Differences between samples could be attributed to plant species, or variations in extraction methods [55,56]. The tannin content remained relatively constant across all samples, ranging narrowly between 0.93 ± 0.0003 and 0.96 ± 0.0007 mg CE/g DW, suggesting that tannin concentration is less influenced by origin or environmental factors compared to phenolics and flavonoids. The tannin content remained relatively constant across all bee pollen samples, varying within a narrow range of 0.93 ± 0.0003 to 0.96 ± 0.0007 mg CE/g DW. This uniformity indicates that tannin accumulation is less affected by the botanical origin or environmental factors compared to total phenolic and flavonoid contents. The slight variations observed among samples—O-04 and M-05 (0.93 ± 0.0003 mg CE/g DW), T-03 and A-04 (0.95 ± 0.0005–0.0006 mg CE/g DW), and G-03 and K-04 (0.96 ± 0.0003–0.0007 mg CE/g DW)—suggest a relatively stable biosynthesis of these condensed polyphenols across different pollen sources. Similar findings were reported by Campos et al. (2010) and Almeida-Muradian et al. (2005) [6,57], who observed that tannin content in bee pollen remains relatively constant regardless of geographic or botanical origin.

### 3.5. Fiber

The total dietary fiber content ranged from 16.27% (O-04) to 28.61% (A-04), highlighting A-04 as the richest sample in fiber, potentially beneficial for digestive health [58]. The fiber content (%) also differed significantly among pollen origins. Samples A-04, M-05, and O-04 exhibited the highest fiber levels, with 28.61 ± 0.89%, 26.06 ± 2.34% and 24.95 ± 2.39% respectively, suggesting potential benefits for digestive health, cholesterol regulation, and metabolic function [59]. G-03 has a moderate fiber content of 20.62 ± 0.13%, while T-03, K-09, and K-04 show the lowest values compared to other pollen samples, 16.27 ± 2.06%, 18.49 ± 1.44% and 18.36 ± 2.22% respectively, indicating a significant variation in dietary fiber availability among pollen sources. The higher fiber content in A-04, M-05, and O-04 makes them promising candidates for functional foods or nutraceutical applications aimed at improving gut health and metabolism. Bee pollen was found to be a good source of total dietary fiber (TDF). Like the findings of Thakur and Nanda [60] most of the TDF in pollen consisted of insoluble dietary fiber (IDF). The TDF content ranged from 17.60% to 31.26%, which is slightly higher than the reported range of 10.6 to 15.9% in other species [60]. Additionally, the soluble dietary fiber (SDF) content varied from 0.86% to 5.92% [61].

### 3.6. Carotenoids and Chlorophyll a and b

Table 4 presents the composition of carotenoids, chlorophyll a, and chlorophyll b in different bee pollen samples from various botanical origins. The content of these compounds varied significantly among the samples. The A-09 sample exhibited the highest levels of carotenoids (87.94 ± 3.2 mg·g^−1^ DW) and chlorophyll a (3.34 ± 0.09 mg·g^−1^ DW) but contained the lowest amount of chlorophyll b (1 ± 0.03 mg·g^−1^ DW), followed by M-05 and O-04. In contrast, the T-03, K-04, and K-09 bee pollen samples had the highest levels of chlorophyll b (1.79 ± 0.02, 1.11 ± 0.04, and 1.48 ± 0.08 mg·g^−1^ DW, respectively) but the lowest concentrations of carotenoids (20.13 ± 3.4, 75.02 ± 2.9, and 62.46 ± 1.88 mg·g^−1^ DW, respectively) and chlorophyll a (1.17 ± 0.05, 2.81 ± 0.04, and 2.63 ± 0.02 mg·g^−1^ DW, respectively) compared to the other bee pollen samples. Notably, the G-03 sample contained high levels of carotenoids (71.49 ± 9.24 mg·g^−1^ DW) and chlorophyll a (2.62 ± 0.1 mg·g^−1^ DW), while its chlorophyll b content was relatively low (0.58 ± 0.03 mg·g^−1^ DW), except when compared to A-09 and M-05. These findings align with those reported by Nguyen et al. (2022), who observed carotenoid levels ranging from 5.88 ± 0.58 to 417.67 ± 9.55 mg·g^−1^ DW, chlorophyll a level between 2.87 ± 0.15 mg·g^−1^ DW and 23.39 ± 10.56 mg·g^−1^ DW, and chlorophyll b levels between 4.96 ± 1.17 mg·g^−1^ DW and 39.17 ± 15.62 mg·g^−1^ DW [62]. Similarly, Muniategui et al. (1990) analysed Spanish bee pollen and reported carotene concentrations ranging from 0.8 to 315.1 mg per 100 g of pollen (dry weight), corresponding to 0.49 to 242.6 mg of carotenoids per gram of bee pollen (dry weight) [63].

### 3.7. Fatty Acid Composition

The fatty acid composition of the seven Tunisian bee pollen (BP) samples analyzed by GC-MS revealed a complex and variable lipid profile, reflecting both the multifloral origin of the pollen and the influence of regional and environmental factors. Sixteen fatty acids were quantified, including nine saturated fatty acids (SFAs) and six unsaturated fatty acids (MUFAs and PUFAs), with total fatty acid content ranging from 0.26 g·100 g^−1^ (sample T-03) to 37.06 g·100 g^−1^ (sample G-03) (Table 5).

SFAs, particularly palmitic acid (C16:0) and lauric acid (C12:0), were consistently detected across all samples, with C16:0 being the most abundant SFA, ranging from 19.62 g·100 g^−1^ to 37.06 g·100 g^−1^. This variability likely arises from differences in floral sources, regional vegetation, and climatic conditions, which are known to modulate pollen lipid composition [35]. Notably, sample G-03 exhibited exceptionally high C16:0 levels, suggesting a predominance of palmitic acid-rich pollen species, consistent with previous reports highlighting the botanical origin as a major determinant of SFA profiles in bee pollen [16].

Fatty acids, including linolenic and palmitic acids, were reported at high concentrations in bee pollen samples [61]. In another study, fatty acids such as lauric acid, palmitic acid, oleic acid and linolenic acid were the major fatty acids found in BP [64]. Monounsaturated fatty acids (MUFAs), primarily oleic acid (C18:1n9c), also varied among samples, ranging from 13.8 g·100 g^−1^ in T-03 to 21.84 g·100 g^−1^ in K-09, whereas minor MUFAs, such as palmitoleic acid (C16:1) and eicosenoid acid (C20:1), were detected only in a few samples (0.34–0.46% in M-05 and A-04) which have likewise been reported as major constituents in *Brassica napus* pollen from India [37]. These MUFAs contribute to the nutritional value and bioactivity of BP, including antioxidant and anti-inflammatory properties [65]. Polyunsaturated fatty acids (PUFAs), notably linoleic acid (C18:2n6) and α-linolenic acid (C18:3n3), were abundant in several samples, with α-linolenic acid reaching up to 31.14 g·100 g^−1^ in A-04. In contrast, Mărgăoan et al. [66] reported that, in 18 bee pollen samples from Turkey and Romania, the concentration of α-linolenic acid was higher than that of linoleic acid. The high n-3 PUFA content suggests potential cardioprotective and anti-inflammatory benefits, in line with prior studies emphasizing the health-promoting properties of n-3-rich bee pollen [67]. However, the low C18:3n3 level in G-03 (1.19 g·100 g^−1^) highlights the impact of dominant floral species, regional vegetation, and post-harvest handling on PUFA content.

Minor fatty acids, including short-chain (C6:0, C8:0, C10:0) and very long-chain fatty acids (C22:0), were present at low concentrations but may still contribute to the overall bioactivity and organoleptic properties of BP. While α-linolenic, palmitic, and oleic acids were identified as the main fatty acids, the lipid extractable fraction of bee pollen cannot be fully characterized solely based on fatty acid composition. Non-polar components such as phospholipids, di- and triglycerides, sterols, and carotenoids also contribute significantly to the nutritional and functional properties of BP [67].

Our results showed that the fatty acid composition of BP may differ depending on the botanical origin of the pollen or the processing and storage conditions. it must be highlighted that the lipid extractable fraction of bee pollen cannot be evaluated only based on the fatty acid composition, since the non-polar fraction may include many other compounds such as phospholipids, di and triglycerides, sterols and carotenoids [68].

Overall, the data indicate that the nutritional and functional quality of Tunisian BP is determined by both dominant and minor fatty acids. Dominant fatty acids define the primary nutritional characteristics, whereas secondary and minor fatty acids enhance the biochemical complexity and potential bioactivity of pollen. This comprehensive fatty acid profiling provides valuable insights for the authentication, valorization, and potential functional food applications of Tunisian bee pollen.

### 3.8. Amino Acid Composition

The analysis of free amino acids revealed notable variability among the seven bee pollen (BP) samples, reflecting differences in their botanical and geographical origins (G-03, O-04, M-05, A-04, T-03, K-04, and K-09) (Table 6), A total of 17 amino acids were identified, with the most abundant being tyrosine, glutamic acid, proline, valine–methionine, tryptophan, cystine, and arginine, depending on the sample. This variability is consistent with previous findings showing that the amino acid composition of BP is highly influenced by the plant species from which the pollen is collected, as well as soil characteristic, beekeeping practices, climatic and environmental factors [6,35]. The amino acids were detected at relatively variant levels across samples; however, significant differences (*p* < 0.05) were observed, reflecting variations in their concentrations among different pollen sources, which ranged from 4.93 ± 0.15 mg·kg^−1^ (sample K-04) to 82.72 ± 2.36 mg·kg^−1^ (sample O-04). Overall, these values are consistent with the total free amino acid content previously reported for commercial bee pollen from Colombia (25.3 ± 1.0 mg/g), Italy (29.4 ± 0.7 mg/g), and Spain (30.8 ± 0.2 mg/g) [69]. Moreover, they exceed those reported for bee pollen originating from single floral sources such as sunflower (12.20 g/100 g) and rape (12.25 g/100 g) [70], highlighting the higher nutritional diversity of multifloral pollen. Among the essential amino acids, tyrosine exhibited the highest concentration across all samples, ranging from 59.52 ± 1.79 mg·kg^−1^ (T-03) to 82.72 ± 2.36 mg·kg^−1^ (O-04). Elevated tyrosine levels suggest the presence of pollen from plant species rich in aromatic amino acids, such as *Brassicaceae* or *Asteraceae* [67]. The high tyrosine content suggests a key role in pollen metabolism and protein synthesis, which may be relevant to its nutritional or functional properties in biological applications [71].

Glutamic acid and aspartic acid, two dominant non-essential amino acids, were also detected at high concentrations, reaching up to 28.3 ± 0.89 mg·kg^−1^ in G-03 and 26.3 ± 0.49 mg·kg^−1^ in O-04, respectively. These amino acids are known to contribute to the umami taste of pollen and play a major role in nitrogen metabolism and protein synthesis [72]. The relatively high content of glutamic acid observed in Tunisian samples aligns with data reported for Spanish and Portuguese bee pollen, where glutamic and aspartic acids were also predominant [66,73].

Essential amino acids such as leucine, valine–methionine, isoleucine, and threonine were found in moderate amounts, contributing to the nutritional value of the samples. In particular, valine–methionine content ranged from 17.30 ± 0.41 mg·kg^−1^ (A-04) to 23.91 ± 0.30 mg·kg^−1^ (M-05), indicating good representation of branched-chain amino acids (BCAAs), which are important for energy metabolism and muscle protein synthesis [74]. The relatively high arginine concentration in M-05 (25.41 ± 0.19 mg·kg^−1^) and A-04 (24.86 ± 0.50 mg·kg^−1^) may be linked to pollen from *Fabaceae* species, which are known to be rich in basic amino acids [75]. Arginine is also known to promote nitric oxide synthesis, supporting vascular and immune functions.

Cystine and tryptophan, present in significant quantities (up to 27.21 ± 0.17 mg·kg^−1^ and 20.90 ± 0.24 mg·kg^−1^, respectively), further enhance the antioxidant potential of BP, as these sulfur-containing and aromatic amino acids can scavenge reactive oxygen species [67]. The detection of glycine, proline, and serine in relatively high amounts suggests their contribution to the structural and metabolic roles in pollen proteins. Proline, which ranged from 13.59 ± 0.37 mg·kg^−1^ (K-09) to 18.66 ± 0.65 mg·kg^−1^ (O-04), is often associated with stress tolerance in plants and with pollen viability [72].

Additionally, as shown in Table 6, sulfur-containing amino acids, such as threonine (10.74–5.84 mg·kg^−1^), were among the least abundant. This has been previously reported and may be linked to the bacterial energy conversion occurring during bee bread fermentation [41,76].

### 3.9. Mineral Composition

The mineral composition of the bee pollen samples is presented in Table 7, offering a comprehensive overview of the concentrations and distribution of essential elements. Nine elements were quantified using the AAS method. The total mineral content of the bee pollen samples ranged from 0.35 ± 0.01 ppm in sample M-05 to 8778.15 ± 470.07 ppm in sample A-04The mineral composition of BP varies significantly (*p* < 0.05) across different origins. Samples O-04, A-04 and M-05 exhibited the highest concentrations of essential minerals, including calcium (Ca) (3172.06 ± 64.18, 2956.20 ± 273.92, and 2910.06 ± 23.59 ppm, respectively), potassium (K) (6323.29 ± 90.85, 7035.93 ± 238.37, and 7502.80 ± 209.18 ppm, respectively), and phosphorus (P) (7573.28 ± 224.32, 8305.16 ± 329.32, and 8778.15 ± 470.07 ppm, respectively), followed by T-03 (2202.94± 44.61, 5419.33 ± 172.2 and, 6137.15 ± 163.26 ppm, respectively), and G-03 (2123.39 ± 81.24, 4429.00 ± 150.78, and 6220.37 ± 543.85 ppm, respectively), (Table 7).

These minerals, ranging between 4000–9000 ppm, are crucial for plant metabolism and human health [77]. Moderate levels of magnesium (Mg) and iron (Fe) (500–2000 ppm) are observed, with Fe being notably higher in A-04 and M-05. Trace elements like zinc (Zn), copper (Cu), manganese (Mn), and molybdenum (Mo) appear in lower concentrations but play key roles in enzymatic functions [78]. K-04 and K-09 have the lowest mineral content, suggesting environmental and genetic influences on mineral accumulation. Similarly, the mineral composition of different BP samples collected from various regions of Greece confirmed the predominance of potassium, calcium, and magnesium [79]. Additionally, previous research has reported findings consistent with our results for these key minerals [61,64,80], though slight variations were observed in iron and copper concentrations. These variations emphasize the potential nutritional value of A-04 and M-05 pollen for dietary and medicinal applications, highlighting the impact of soil composition, environmental conditions, and plant species differences on mineral uptake [64,79]. In addition, Silvia Valverde et al. (2023) found that bee pollen mineral content is significantly influenced by geographic and edaphic factors, including soil nutrient availability and local botanical origins [81].

Globally, bee pollen is commonly reported to contain essential minerals such as calcium (Ca), copper (Cu), chromium (Cr), iron (Fe), potassium (K), magnesium (Mg), manganese (Mn), sodium (Na), phosphorus (P), and zinc (Zn). In contrast, trace elements like boron (B), molybdenum (Mb), and selenium (Se) are rarely detected and are present only in pollen from certain regions, with concentrations ranging from 8.2–14 mg/kg for B, 0.1–4.6 mg/kg for Mb, and less than 0.01–4.5 mg/kg for Se [82,83].

**Figure 3:**
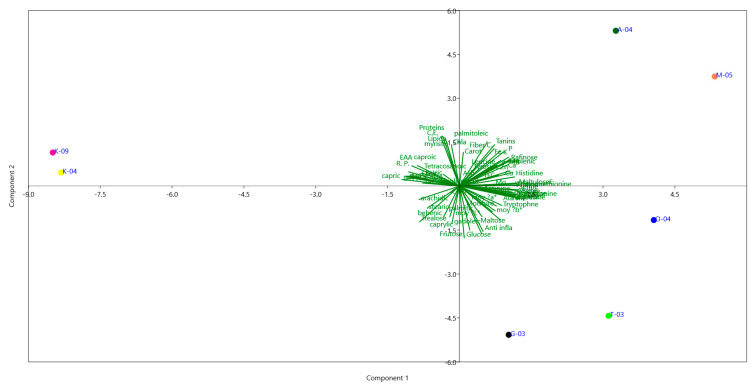
Loading plot of the principal component analysis (PCA) of variations in pollen biochemical composition across regions.

This study provides, for the first time, a comprehensive biochemical and multivariate characterization of pollen collected from diverse Tunisian regions. PCA loadings indicated that PC1, explaining 49.43% of the variance, was mainly associated with sugars (e.g., maltulose, turanose, trehalose), amino acids (e.g., serine, glutamic acid, proline), minerals (Zn, Cu, Mg, Ca, P), and selected fatty acids (capric, oleic, linoleic acids). PC2 (21.52% variance) was influenced by lipids, proteins, and antioxidant indicators (TFC, chlorophyll b), highlighting a secondary gradient of variation. PC3 (10.89%) reflected contributions from moisture, ash, lauric acid, and colour parameters, representing more subtle differences among samples. Together, these components captured the main patterns of variation in pollen biochemical composition across regions (Figure 3).

The PCA clearly distinguished samples according to regional origin, reflecting pronounced environmental and metabolic differentiation. Pollen from Aousja (A-04) and Mateur (M-05), located in the humid north, was positioned on the positive side of PC1 and characterized by higher lipid content, oleic and linoleic acids, phenolics, flavonoids, and antioxidant activities (DPPH, ABTS, FRAP), indicating a richer nutritional and bioactive profile. Om Heni (O-04) pollen showed an intermediate composition, with moderate levels of proteins, essential amino acids, and sugars, suggesting a balanced metabolic status. In contrast, pollen from Kairouan (K-04, K-09) in the semi-arid center was separated on the negative side of PC1, with lower lipids and antioxidants but relatively higher ash and mineral contents (Ca, Mg, Fe), likely reflecting adaptation to drier conditions. Southern Tozeur (T-03) pollen was differentiated along PC2 by elevated amino acids (glutamic acid, histidine, arginine) and soluble sugars (glucose, fructose, trehalose), pointing to osmoprotective adaptations to arid environments. Pollen from Nabeul (G-03), from the subhumid coast, displayed distinctive associations with polyunsaturated fatty acids and protein-derived metabolites, suggesting a metabolically balanced composition.

Overall, these findings reveal that Tunisian pollens exhibit region-specific biochemical fingerprints shaped by agro-climatic gradients, from humid to arid zones. The integration of primary metabolites (sugars, amino acids, fatty acids), minerals, and antioxidant indicators provides novel evidence of how environmental factors modulate pollen quality and functionality. This study thus highlights pollen’s potential as both a functional food ingredient and a biochemical marker of geographical origin and ecological adaptation [84].

## 4. Conclusions

This study provides for the first a comprehensive characterization of seven multifloral bee pollen samples from different Tunisian regions: Bizerte-Aousja (A-04), Nabeul-Ghardiai (G-03), Kairouan (K-04), Kairouan-Weslatia (K-09), Bizerte-Mateur (M-05), Bizerte-Om Heni (O-04), and Tozeur (T-03). Significant regional variations were observed across nutritional and bioactive components. Bee pollen from the northern regions, particularly Bizerte-Aousja (A-04) and Bizerte-Mateur (M-05), exhibited the highest protein, dietary fiber, carotenoids, chlorophylls, phenolic compounds, amino acid, and mineral contents, indicating superior nutritional and functional potential. Samples from Kairouan (K-04 and K-09) in the southern region were richer in lipids, unsaturated fatty acids reflecting adaptations to local floral and climatic conditions. This work represents the first integrative comparative study of Tunisian multifloral bee pollen, providing valuable insights for its nutraceutical and functional food applications, and highlighting northern regions as optimal sources of high-quality pollen.

## Figures and Tables

**Table 1:** Chemical and nutritional composition of BP samples.

	pH	Water Content	Ash Content	Total Lipid Content	Protein Content	Total Dietary Fiber	Total Carbohydrates	Energy
Samples		(%)	(%)	(%)	(%)	(%)	(g/100 g)	(kcal/100 g DW)
**G-03**	4.79 ± 0.02	8.61 ± 0.21 ^d^	2.21 ± 0.10	1.60 ± 0.03	17.21 ± 3.23	20.63 ± 0.13	58.4 ± 3.4	357.9 ± 0.2
**T-03**	4.81 ± 0.01	9.14 ± 0.20 ^cd^	2.04 ± 0.36	2.30 ± 0.11 ^f^	19.07 ± 2.64	24.96 ± 2.39	62.7 ± 3.5	370.9 ± 8.5
**O-04**	4.54 ± 0.04	9.53 ± 0.11 ^cd^	2.21 ± 0.37	2.46 ± 0.01	16.35 ± 0.75	16.27 ± 2.06	51.6 ± 1.1	353.4 ± 7.3
**M-05**	4.61 ± 0.02	8.93 ± 0.35 ^d^	2.78 ± 0.07	4.43 ± 0.02 ^ab^	21.21 ± 0.49	26.07 ± 2.35 a	45.5 ± 3.8	358.9 ± 8.2
**A-04**	4.79 ± 0.03	6.95 ± 0.08	2.00 ± 0.05	3.85 ± 0.96 ^ab^	22.19 ± 0.65	28.61 ± 0.89 ^ade^	43.4 ± 2.8	354.0 ± 8.3
**K-09**	5.46 ± 0.05	8.35 ± 0.29 ^d^	2.34 ± 0.28	4.05 ± 0.16 ^ab^	22.47 ± 0.62	18.50 ± 1.44	52.6 ± 3.3	373.9 ± 3.8
**K-04**	5.15 ± 0.08	6.03 ± 0.06	1.89 ± 0.04	3.46 ± 0.33 ^ab^	19.79 ± 0.87	18.36 ± 2.23	56.5 ± 4.4	373.1 ± 9.9

Data presented as mean SEM of three independent assays. ^a^ *p* < 0.05, vs. O-04, ^b^ *p* < 0.05 vs. G-03, ^c^ *p* < 0.05 vs. A-04, ^d^ *p* < 0.05 vs. K-04, ^e^ *p* < 0.05 vs. K-09, and ^f^ *p* < 0.05 vs. K-09.

**Table 2:** Phytochemical analysis of BP samples.

Samples	Tannins	Phlobotannins	Terpenoids	Alkaloids	Steroids	Saponins
**T-03**	+	+	+	+	++	+
**K-09**	+	++	+	+	++	+
**K-04**	++	+	+	+	+	+
**M-05**	+	+	+	+	+	+
**A-04**	+++	+++	++	++	+++	+
**O-04**	++	+	+	+	+	+
**G-03**	+	+	+	+	++	+

**Table 3:** Tannins, total flavonoid and phenolic content and nutritional composition of BP samples.

	Total Phenolic Content	Total Flavonoid Content	Tannins
	mg GAE·g DW	mg QE/g DW	mg CE·G DW
**G-03**	4.79 ± 0.02	3.49 ± 0.002	0.96 ± 0.0007
**T-03**	4.81 ± 0.01	3.50 ± 0.002	0.95 ± 0.0006
**O-04**	4.54 ± 0.04	3.37 ± 0.001	0.93 ± 0.0003
**M-05**	4.61 ± 0.02	3.51 ± 0.001	0.93 ± 0.0003
**A-04**	4.79 ± 0.03	3.44 ± 0.005	0.95 ± 0.0005
**K-09**	5.46 ± 0.05	3.52 ± 0.002	0.95 ± 0.0005
**K-04**	5.15 ± 0.08	3.27 ± 0.2	0.96 ± 0.0003

Data presented as mean SEM of three independent assays.

**Table 4:** Photosynthetic pigments composition of BP samples.

	Chlorophyll		Carotenoids
	**a**	b	
	mg·g^−1^		µg·g^−1^
**G-03**	2.63 ± 0.11 ^a^	0.59 ± 0.09	71.37 ± 9.25 ^a^
**T-03**	2.43 ± 0.54 ^a^	1.24 ± 0.43 ^b^	84.71 ± 13.12 ^a^
**O-04**	1.18 ± 0.15	1.79 ± 0.23 ^b^	20.14 ± 3.40
**M-05**	2.90 ± 0.16 ^a^	1.00 ± 0.34 ^ab^	82.79 ± 8.08 ^a^
**A-04**	3.35 ± 0.10 ^a^	1.01 ± 0.38 ^ab^	87.94 ± 3.20 ^a^
**K-09**	2.64 ± 0.23 ^a^	1.48 ± 0.18 ^b^	62.46 ± 1.88 ^a^
**K-04**	2.82 ± 0.15 ^a^	1.12 ± 0.41 ^ab^	75.03 ± 2.98 ^a^

Data presented as mean SEM of three independent assays. ^a^ *p* < 0.05, vs. O-04, ^b^ *p* < 0.05 vs. G-03.

**Table 5:** Fatty acid composition of the BP samples (g·100 g^−1^).

	A-04	G-03	K-04	K-09	M-05	T-03	O-04
**C6:0**	0.541 ± 0.03 ^abe^	ND	0.567 ± 0.02 ^abe^	1.85 ± 0.39 ^ab^	0.38 ± 0.06 ^abe^	0.26 ± 0.02 ^abef^	ND
**C8:0**	0.38 ± 0.03 ^ab^	6.59 ± 0.24 ^a^	1.03 ± 0.04 ^abc^	2.46 ± 0.37 ^abc^	0.73 ± 0.05 ^abc^	2.38 ± 0.21 ^abcf^	ND
**C10:0**	0.76 ± 0.03 ^a^	1.14 ± 0.02 ^a^	3 ± 0.13 ^ac^	2.68 ± 0.09 ^ac^	0.48 ± 0.07 ^abde^	0.27 ± 0.01 ^acf^	ND
**C12:0**	9.07 ± 0.55	7.05 ± 0.21	12.75 ± 0.91 ^abe^	5.5 ± 0.3	3.03 ± 0.31 ^abcde^	4.23 ± 0.45 ^abcd^	1.43 ± 0.24 ^bcde^
**C14:0**	11.08 ± 1.05 ^ab^	3.62 ± 0.14	8.67 ± 0.27 ^abe^	5.33 ± 0.64	5.46 ± 0.04	2.43 ± 0.23 ^acdef^	4.47 ± 0.47
**C16:0**	20.46 ± 0.85 ^b^	37.06 ± 0.27	24.05 ± 1.47	22.58 ± 1.7 ^b^	19.62 ± 1.96 ^b^	19.71 ± 0.66 ^b^	21 ± 0.9 ^b^
**C16:1**	0.47 ± 0.06 ^de^	ND	ND	ND	0.34 ± 0.04 ^abde^	ND	ND
**C18:0**	1.69 ± 0.12 ^abde^	8.02 ± 0.06	5.76 ± 0.16	10.11 ± 1.08 ^d^	5.97 ± 0.14 ^ce^	5.18 ± 0.09 ^ce^	5.44 ± 0.06
**C18:1n9c**	17.27 ± 0.31	16.25 ± 1.38 ^aced^	21.43 ± 0.9	21.84 ± 1.5	16.07 ± 0.66	13.8 ± 0.17 ^abcde^	21.71 ± 0.7
**C18:2n6c**	4.69 ± 0.47 ^a^	ND	5.3 ± 0.41 ^a^	5.59 ± 0.55 ^a^	7.71 ± 0.21 ^ac^	8.17 ± 0.36 ^ac^	15.55 ± 0.43
**C18:3n3**	31.14 ± 0.55 ^bd^	1.19 ± 0.27	5.97 ± 0.1 ^b^	10.12 ± 0.88 ^bd^	30.93 ± 1.13 ^bde^	27.97 ± 0.54 ^bd^	25.59 ± 1.25 ^bd^
**C20:0**	0.91 ± 0.1 ^be^	2.86 ± 0.00	2.28 ± 0.18	5.12 ± 0.83	2.01 ± 0.05 ^e^	2.39 ± 0.23 ^e^	1.54 ± 0.28 ^e^
**C20:1**	0.99 ± 0.07	11.02 ± 0.22 ^acde^	2.06 ± 0.13	1.2 ± 0.01	1.9 ± 0.11 ^abcd^	9.84 ± 0.52 ^acedf^	ND
**C22:0**	1.23 ± 0.07 ^ade^	2.62 ± 0.39 ^c^	2.61 ± 0.27	2 ± 0.21	1.11 ± 0.08 ^ade^	1.69 ± 0.15	3.28 ± 0.36
**C22:1n9**	0.28 ± 0.01 ^abde^	ND	2.26 ± 0.01	0.97 ± 0.05 ^d^	ND	ND	ND
**C24:0**	1.45 ± 0.16 ^a^	2.84 ± 0.52 ^ac^	2.12 ± 0.15 ^ac^	3.32 ± 0.68 ^ac^	3.95 ± 0.23 ^ac^	1.42 ± 0.16 ^bef^	ND

Data presented as mean SEM of three independent assays. ^a^ *p* < 0.05, vs. O-04, ^b^ *p* < 0.05 vs. G-03, ^c^ *p* < 0.05 vs. A-04, ^d^ *p* < 0.05 vs. K-04, ^e^ *p* < 0.05 vs. K-09, and ^f^ *p* < 0.05 vs. K-09.

**Table 6:** Free amino acid composition of the BP samples (mg·kg^−1^).

	G-03	O-04	M-05	A-04	T-03	K-04	K-09
**Aspartic acid**	26.06 ± 0.27	23.92 ± 0.95	24.62 ± 0.46 ^de^	24.77 ± 0.33	18 ± 0.19 ^bf^	15.9 ± 0.32 ^abc^	14.75 ± 0.55 ^abc^
**Glutamic acid**	28.3 ± 0.89	26.53 ± 0.49	27.35 ± 0.84 ^de^	27.44 ± 0.15	19.99 ± 0.57 ^abcdef^	12.04 ± 0.63 ^abc^	13.16 ± 0.48 ^abc^
**Histidine**	8.72 ± 0.58	10.14 ± 0.39	12.53 ± 0.27 ^bde^	11.22 ± 0.71	8.04 ± 0.34	6.21 ± 0.40	7.15 ± 0.14
**Serine**	14.26 ± 0.88	15.43 ± 1.31	15.67 ± 0.32 ^de^	14.75 ± 0.42	12.29 ± 0.41 ^e^	9.3 ± 0.24 ^abc^	7.44 ± 0.37 ^abc^
**Proline**	17.16 ± 0.8	18.66 ± 0.65	19.16 ± 0.32 ^de^	17.37 ± 0.22	14.25 ± 0.33 ^abc^	14.77 ± 0.23 ^abc^	13.59 ± 0.37 ^abc^
**Threonine**	8.39 ± 0.18	10.11 ± 0.45	10.75 ± 0.55 ^de^	9.32 ± 0.27	5.84 ± 0.13 ^af^	4.93 ± 0.15 ^abc^	6.35 ± 0.10 ^acf^
**Glycine**	12.72 ± 0.37	14.55 ± 0.38	14.16 ± 0.33	13.42 ± 0.25	9.89 ± 0.23 ^f^	7.64 ± 0.25 ^abcf^	5.64 ± 0.37 ^abcf^
**Arginine**	23.77 ± 1.09	8.71 ± 0.15 ^bcdef^	25.41 ± 0.19	24.86 ± 0.50	19.25 ± 0.26	18.1 ± 0.23 ^cf^	23.21 ± 0.23
**Alanine**	14.15 ± 0.30	11.84 ± 0.26	12.47 ± 0.74	12.11 ± 0.81	7.22 ± 0.20 ^b^	6.41 ± 0.22 ^abcf^	5.97 ± 0.42 ^abcf^
**Tyrosine**	74.33 ± 2.49 ^d^	82.72 ± 2.36 ^de^	81.47 ± 0.54 ^de^	79.44 ± 0.68 ^e^	59.52 ± 1.79 ^abcdef^	68.82 ± 1.83	78.53 ± 1.74
**Phenylalanine**	10.62 ± 0.37	13.06 ± 0.53 ^de^	11.2 ± 0.72	13.66 ± 0.36 ^de^	7.67 ± 0.12 ^ac^	8.57 ± 0.28	7.62 ± 0.38
**Isoleucine**	12.83 ± 0.46	11.96 ± 0.60	11.99 ± 0.02	11.76 ± 0.35	9.49 ± 0.15	8.00 ± 0.27	7.03 ± 0.35 ^abcf^
**Leucine**	15.46 ± 0.10	17.88 ± 0.09	21.82 ± 0.33	17.78 ± 0.41	13.05 ± 0.34 ^acf^	17.39 ± 0.53	15.21 ± 0.24
**Valine-methionine**	20.67 ± 0.44	21.24 ± 1.27 ^e^	23.91 ± 0.30 ^de^	21.74 ± 0.30 ^e^	17.14 ± 0.41 ^f^	18.44 ± 0.27	15.7 ± 0.38
**Tryptophan**	18.34 ± 0.25	20.19 ± 0.53	15.3 ± 0.19	20.9 ± 0.24	11.36 ± 0.23 ^abc^	14.7 ± 0.35 ^ac^	13.11 ± 0.61 ^abc^
**Cystine**	25.22 ± 0.64 ^de^	26.1 ± 0.11 ^de^	27.21 ± 0.17 ^de^	26.1 ± 0.43	20.11 ± 0.21 ^abcf^	20.34 ± 0.64	16.94 ± 0.22
**Lysine**	15.59 ± 0.34 ^de^	13.83 ± 0.15 ^e^	14.87 ± 0.11 ^de^	13.03 ± 0.26	9.58 ± 0.18	9.13 ± 0.12 ^f^	7.74 ± 0.15 ^fc^

Data presented as mean SEM of three independent assays. ^a^ *p* < 0.05, vs. O-04, ^b^ *p* < 0.05 vs. G-03, ^c^ *p* < 0.05 vs. A-04, ^d^ *p* < 0.05 vs. K-04, ^e^ *p* < 0.05 vs. K-09, and ^f^ *p* < 0.05 vs. K-09.

**Table 7:** Mineral composition of the BP samples (ppm).

	G-03	O-04	M-05	A-04	T-03	K-04	K-09
**Zn**	62.73 ± 5.73	69.31 ± 1.85	70.37 ± 2.9	69.88 ± 0.24	50.19 ± 2.78 ^abcf^	38.53 ± 6.09 ^abcf^	46.08 ± 3.03 ^abcf^
**Cu**	21.04 ± 0.86 ^de^	24.00 ± 0.35 ^de^	25.10 ± 2.89 ^de^	23.68 ± 0.8 ^de^	17.01 ± 0.41 ^abcf^	13.09 ± 0.48	12.77 ± 0.18
**Mn**	111.23 ± 5.68 ^de^	147.27 ± 6.44 ^dee^	153.86 ± 7.5 ^de^	151.64 ± 8.54 ^de^	106.69 ± 8.74 ^abcf^	90.26 ± 4.89	86.85 ± 6.4
**Fe**	86.53 ± 5.13 ^d^	145.92 ± 2.32 ^de^	148.61 ± 4.86 ^de^	152.44 ± 8.68 ^de^	110.10 ± 6.5 ^bcf^	98.76 ± 4.95	94.32 ± 2.49
**Mg**	2122.34 ± 71.7	2853.21 ± 11.21	2877.29 ± 342.95	3016.96 ± 288.35	2106.05 ± 74.24 ^abcf^	1657.89 ± 128.84 ^abcf^	1788.57 ± 224.42 ^abcf^
**K**	4429.00 ± 150.78 ^de^	6323.29 ± 90.85 ^de^	7035.93 ± 238.37 ^de^	7502.80 ± 209.18 ^de^	5419.33 ± 172.29 ^abcf^	4249.29 ± 157.65	4517.29 ± 101.26
**Ca**	2123.39 ± 81.24 ^de^	3172.06 ± 64.18 ^de^	2956.20 ± 273.92 ^de^	2910.06 ± 23.59 ^de^	2202.94 ± 44.61 ^abcf^	1673.35 ± 107.06	1932.24 ± 30.57
**P**	6220.37 ± 543.85	7573.28 ± 224.32	8305.16 ± 329.32	8778.15 ± 470.07	6137.15 ± 163.26 ^acf^	4919.37 ± 114.82 ^abcf^	5202.07 ± 137.97 ^abcf^
**Mo**	0.64 ± 0.08 ^def^	0.47 ± 0.04 ^de^	0.35 ± 0.01 ^de^	0.51 ± 0.04 ^de^	0.07 ± 0.01 ^abcf^	0.11 ± 0.02	0.05 ± 0.01

Data presented as mean SEM of three independent assays. ^a^ *p* < 0.05, vs. O-04, ^b^ *p* < 0.05 vs. G-03, ^c^ *p* < 0.05 vs. A-04, ^d^ *p* < 0.05 vs. K-04, ^e^ *p* < 0.05 vs. K-09, and ^f^ *p* < 0.05 vs. K-09.

## Data Availability

The data presented in this study are available from the corresponding authors upon reasonable request due to legal restrictions. As the dataset is part of an ongoing project, it is not yet finalized for public.

## Supplementary Materials

The following supporting information can be downloaded at: https://www.mdpi.com/article/10.3390/foods14233986/s1, Table S1. Geographical and bioclimatic characteristics of the seven bee pollen collection sites in Tunisia; Table S2. Fatty acids identified by GC-MS on bee pollen samples from Tunisia; Table S3. Amino Acids identified by HPLC-FLD on bee pollen samples from Tunisia.

## Abbreviations

The following abbreviations are used in this manuscript:

ABPE	Aqueous bee pollen extract
DW	dry weight
CRD	Completely randomized design

## References

[1] Bobiş O., Mărghitaş L.A., Dezmirean D., Morar O., Bonta V., Chirilă F. (2010). Quality Parameters and Nutritional Value of Different Commercial Bee Products. Bull. UASVM Anim. Sci. Biotechnol..

[2] Di Chiacchio I.M., Paiva I.M., de Abreu D.J., Carvalho E.E., Martínez P.J., Carvalho S.M., Mulero V., Murgas L.D.S. (2021). Bee Pollen as a Dietary Supplement for Fish: Effect on the Reproductive Performance of Zebrafish and the Immunological Response of Their Offspring. Fish Shellfish Immunol..

[3] Bogdanov S. (2011). The Bee Pollen Book. Bulg. Bee Prod. Sci..

[4] Komosinska-Vassev K., Olczyk P., Kaźmierczak J., Mencner L., Olczyk K. (2015). Bee Pollen: Chemical Composition and Therapeutic Application. Evid.-Based Complement. Altern. Med..

[5] Rodríguez-Flores M.S., Escuredo O., Seijo M.C., Rojo S., Vilas-Boas M., Falcão S.I. (2023). Phenolic Profile of Castanea Bee Pollen from the Northwest of the Iberian Peninsula. Separations.

[6] de Almeida-Muradian L.B., Pamplona L.C., Coimbra S., Barth O.M. (2005). Chemical Composition and Botanical Evaluation of Dried Bee Pollen Pellets. J. Food Compos. Anal..

[7] Campos M.G.R., Bogdanov S., De Almeida-Muradian L.B., Szczesna T., Mancebo Y., Frigerio C., Ferreira F. (2008). Pollen Composition and Standardisation of Analytical Methods. J. Apic. Res..

[8] Adaškevičiūtė V., Kaškonienė V., Kaškonas P., Barčauskaitė K., Maruška A. (2019). Comparison of Physicochemical Properties of Bee Pollen with Other Bee Products. Biomolecules.

[9] Kostić A.Ž., Milinčić D.D., Barać M.B., Ali Shariati M., Tešić Ž.L., Pešić M.B. (2020). The Application of Pollen as a Functional Food and Feed Ingredient—The Present and Perspectives. Biomolecules.

[10] Nayik G.A., Shah T.R., Muzaffar K., Wani S.A., Gull A., Majid I., Bhat F.M. (2014). Honey: Its History and Religious Significance: A Review. Univers. J. Pharm..

[11] Otmani A., Amessis-Ouchemoukh N., Mouhoubi-Tafinine Z., Tighlit K., Redouan I., Terrab A., Ouchemoukh S. (2022). Contribution of Organic Bee Pollen to the Determination of Botanical Origin of Honey and Its Impact on Its Biological Properties. CBC.

[12] Zuluaga C.M., Serratob J.C., Quicazana M.C. (2015). Chemical, Nutritional and Bioactive Characterization of Colombian Bee-Bread. Chem. Eng..

[13] Bayram N.E., Gercek Y.C., Celik S., Mayda N., Kostić A.Ž., Dramićanin A.M., Özkök A. (2021). Phenolic and Free Amino Acid Profiles of Bee Bread and Bee Pollen with the Same Botanical Origin–Similarities and Differences. Arab. J. Chem..

[14] Soares de Arruda V.A., Vieria dos Santos A., Figueiredo Sampaio D., da Silva Araujo E., de Castro Peixoto A.L., Estevinho L.M., de Almeida-Muradian L.B. (2021). Brazilian bee pollen: Phenolic content, antioxidant properties and antimicrobial activity. J. Apic. Res..

[15] De-Melo A.A.M., de Almeida-Muradian L.B. (2017). Chemical composition of bee pollen. Bee Products—Chemical and Biological Properties.

[16] Pascoal A., Rodrigues S., Teixeira A., Feás X., Estevinho L.M. (2014). Biological activities of commercial bee pollens: Antimicrobial, antimutagenic, antioxidant and anti inflammatory. Food Chem. Toxicol..

[17] Abdelkader F.B. (2020). Situation of Beekeeping in North Africa. J. Apitherapy Nat..

[18] Jribi S., Hanafi N.-E.H., Jmal S., Salem H.B., Ismail H.B., Debbabi H. (2024). An Insight into Preference, Quality Perception and Attitudes Towards Honey Consumption in Tunisia.

[19] Sakraoui A., Arrari F., Sakhraoui A., Mejri M. (2025). Ethno-Pharmacological Survey on the Traditional Use of Bee Pollen in Tunisia. J. Nutraceuticals Health.

[20] Jmal S., Ben Ismail H., Hamzaoui M., Ben Salem H., Debbabi H. (2024). Analysis of Tunisian Beekeepers’ Perceptions in Honey Bee Diseases and Pests Management. Egypt. J. Agric. Sci..

[21] Ouertani E., Erraach Y., Arfa L., Kallas Z., De Magistris T., Ornelas Herrera S.I. (2025). Beekeepers’ Intentions to Adopt Resilience Strategies for Climate Change: A Comparative and Integrated Approach Using Theory of Planned Behavior and Protection Motivation Theory. Front. Clim..

[22] Campos M., Anjos O., Chica M., Campoy P., Nôžková J., Almaraz-Abarca N., Barreto L., Nordi J., Estevinho L., Pascoal A. (2021). Standard Methods for Pollen Research. J. Apic. Res..

[23] De Novais J.S., Absy M.L., dos Santos F. (2014). de A.R. Pollen Types Collected by Tetragonisca Angustula (Hymenoptera: Apidae) in Dry Vegetation in Northeastern Brazil. Eur. J. Entomol..

[24] Cunniff P., Washington D. (1997). Official Methods of Analysis of AOAC International. J. AOAC Int..

[25] Carocho M., Morales P., Ciudad-Mulero M., Fernandez-Ruiz V., Ferreira E., Heleno S., Rodrigues P., Barros L., Ferreira I.C. (2020). Comparison of Different Bread Types: Chemical and Physical Parameters. Food Chem..

[26] Trease G., Evans M. (1989). Text Book of Pharmacognosy.

[27] Singleton V. (1999). Analysis of Total Phenols and Other Oxidation Substrates and Antioxidants by Means of Folin-Ciocalteu Reagent. Methods Enzymol..

[28] Price M.L., Van Scoyoc S., Butler L.G. (1978). A Critical Evaluation of the Vanillin Reaction as an Assay for Tannin in Sorghum Grain. J. Agric. Food Chem..

[29] Zhishen J., Mengcheng T., Jianming W. (1999). The Determination of Flavonoid Contents in Mulberry and Their Scavenging Effects on Superoxide Radicals. Food Chem..

[30] Séraphin D.K., Youssouf K.K., Doudjo S., Emmanuel A.N., Benjamin Y.K., Dago G. (2015). Caractérisation Biochimique et Fonctionnelle Des Graines de Sept Cultivars de Voandzou [*Vigna subterranea* (L.) Verdc. Fabaceae] Cultivés En Côte d’Ivoire. Eur. Sci. J..

[31] Lichtenthaler H.K., Wellburn A.R. (1985). Determination of Total Cartionoids and Chlorophyll A and B of Leaf in Different Solvents. Biochem. Soc. Trans..

[32] Ng V., Cribbie R. (2017). Modeling Continuous, Skewed and Heteroscedastic Outcomes in Psychology: Is Generalized Modeling the Best’fit’?.

[33] Hammer Ø., Harper D.A.T. (2012). PAST Paleontological Statistics Version 2.17 Reference Manual.

[34] Aylanc V., Tomás A., Russo-Almeida P., Falcão S.I., Vilas-Boas M. (2021). Assessment of Bioactive Compounds under Simulated Gastrointestinal Digestion of Bee Pollen and Bee Bread: Bioaccessibility and Antioxidant Activity. Antioxidants.

[35] Oroian M., Dranca F., Ursachi F. (2022). Characterization of Romanian Bee Pollen—An Important Nutritional Source. Foods.

[36] Thakur M., Nanda V. (2021). Screening of Indian Bee Pollen Based on Antioxidant Properties and Polyphenolic Composition Using UHPLC-DAD-MS/MS: A Multivariate Analysis and ANN Based Approach. Food Res. Int..

[37] Thakur M., Nanda V. (2018). Assessment of Physico-Chemical Properties, Fatty Acid, Amino Acid and Mineral Profile of Bee Pollen from India with a Multivariate Perspective. J. Food Nutr. Res..

[38] Dranca F., Ursachi F., Oroian M. (2020). Bee Bread: Physicochemical Characterization and Phenolic Content Extraction Optimization. Foods.

[39] Isopescu R.D., Spulber R., Josceanu A.M., Mihaiescu D.E., Popa O. (2020). Romanian bee pollen classification and property modelling. J. Apic. Res..

[40] Oroian M., Ursachi F., Dranca F. (2020). Ultrasound-Assisted Extraction of Polyphenols from Crude Pollen. Antioxidants.

[41] Kieliszek M., Piwowarek K., Kot A.M., Błażejak S., Chlebowska-Śmigiel A., Wolska I. (2018). Pollen and Bee Bread as New Health-Oriented Products: A Review. Trends Food Sci. Technol..

[42] Khalifa S.A., Elashal M.H., Yosri N., Du M., Musharraf S.G., Nahar L., Sarker S.D., Guo Z., Cao W., Zou X. (2021). Bee Pollen: Current Status and Therapeutic Potential. Nutrients.

[43] Li Q., Liang X., Zhao L., Zhang Z., Xue X., Wang K., Wu L. (2017). UPLC-Q-Exactive Orbitrap/MS-Based Lipidomics Approach to Characterize Lipid Extracts from Bee Pollen and Their in Vitro Anti-Inflammatory Properties. J. Agric. Food Chem..

[44] Tomás A., Falcão S.I., Russo-Almeida P., Vilas-Boas M. (2017). Potentialities of Beebread as a Food Supplement and Source of Nutraceuticals: Botanical Origin, Nutritional Composition and Antioxidant Activity. J. Apic. Res..

[45] Martins M.C. (2011). Physicochemical Composition of Bee Pollen from Eleven Brazilian States.

[46] Gardana C., Del Bo C., Quicazán M.C., Corrrea A.R., Simonetti P. (2018). Nutrients, Phytochemicals and Botanical Origin of Commercial Bee Pollen from Different Geographical Areas. J. Food Compos. Anal..

[47] Venkataramaiah C.H., Wudayagiri R. (2013). Phytochemical Screening of Bioactive Compounds Present in the Seed of *Celastrus Paniculatus* Role in Traditional Medicine. Seas. Indo Amer. J. Pharm. Res..

[48] Xiao M., Li S., Pei W., Gu Y., Piao X. (2025). Natural Saponins on Cholesterol-Related Diseases: Treatment and Mechanism. Phytother. Res..

[49] Kaur J., Rasane P., Kumar V., Nanda V., Bhadariya V., Kaur S., Singh J. (2024). Exploring the Health Benefits of Bee Pollen and Its Viability as a Functional Food Ingredient. Rev. Agric. Sci..

[50] Djelloul Z. (2025). Physicochemical Characterization and Antioxidant Activity of Algerian Bee Pollens: Exploring Geographical Influences. Curr. Nutr. Food Sci..

[51] Crozier A., Jaganath I.B., Clifford M.N. (2009). Dietary Phenolics: Chemistry, Bioavailability and Effects on Health. Nat. Prod. Rep..

[52] De-Melo A.A.M., Estevinho L.M., Moreira M.M., Delerue-Matos C., Freitas A.D.S.D., Barth O.M., Almeida-Muradian L.B.D. (2018). Phenolic Profile by HPLC-MS, Biological Potential, and Nutritional Value of a Promising Food: Monofloral Bee Pollen. J. Food Biochem..

[53] Mustafa R.A., Hamid A.A., Mohamed S., Bakar F.A. (2010). Total Phenolic Compounds, Flavonoids, and Radical Scavenging Activity of 21 Selected Tropical Plants. J. Food Sci..

[54] Chiva-Blanch G., Visioli F. (2012). Polyphenols and Health: Moving beyond Antioxidants. J. Berry Res..

[55] Azmir J., Zaidul I.S.M., Rahman M.M., Sharif K.M., Mohamed A., Sahena F., Jahurul M.H.A., Ghafoor K., Norulaini N.A., Omar A.K. (2013). Techniques for Extraction of Bioactive Compounds from Plant Materials: A Review. J. Food Eng..

[56] Rodríguez De Luna S.L., Ramírez-Garza R.E., Serna Saldívar S.O. (2020). Environmentally Friendly Methods for Flavonoid Extraction from Plant Material: Impact of Their Operating Conditions on Yield and Antioxidant Properties. Sci. World J..

[57] Campos M.G.R., Frigerio C., Lopes J., Bogdanov S. (2010). What Is the Future of Bee-Pollen. J. ApiProd. ApiMed. Sci..

[58] Arrari F., Jabri M.-A., Hammami I., Sebai H. (2022). Extraction of Pectin from Orange Peel and Study of Its Protective Effect Against Loperamide-Induced Impaired Gastrointestinal Motor Functions and Oxidative Stress in Rats. J. Med. Food.

[59] Lattimer J.M., Haub M.D. (2010). Effects of Dietary Fiber and Its Components on Metabolic Health. Nutrients.

[60] Thakur M., Nanda V. (2020). Composition and Functionality of Bee Pollen: A Review. Trends Food Sci. Technol..

[61] Yang K., Wu D., Ye X., Liu D., Chen J., Sun P. (2013). Characterization of Chemical Composition of Bee Pollen in China. J. Agric. Food Chem..

[62] Nguyen H.C., Liang-Chih L.I.U., Ming-Cheng W.U., Tai-Pei L.I.N., Chiou-Ying Y., Huang M.-Y. (2022). Chemical Constituents, Antioxidant, and Anticancer Activities of Bee Pollen from Various Floral Sources in Taiwan. Not. Bot. Horti Agrobot. Cluj-Napoca.

[63] Muniategui S., Sancho M.T., López J., Huidobro J.F., Simal J. (1990). Determination of Carotenes from Bee-Collected Pollen by High Performance Liquid Chromatography. J. Apic. Res..

[64] Mayda N., Özkök A., Ecem Bayram N., Gerçek Y.C., Sorkun K. (2020). Bee Bread and Bee Pollen of Different Plant Sources: Determination of Phenolic Content, Antioxidant Activity, Fatty Acid and Element Profiles. Food Meas..

[65] Viuda-Martos M., Ruiz-Navajas Y., Fernández-López J., Pérez-Alvarez J.A. (2008). Functional Properties of Honey, Propolis, and Royal Jelly. J. Food Sci..

[66] Mărgăoan R., Stranț M., Varadi A., Topal E., Yücel B., Cornea-Cipcigan M., Campos M.G., Vodnar D.C. (2019). Bee Collected Pollen and Bee Bread: Bioactive Constituents and Health Benefits. Antioxidants.

[67] Feás X., Vázquez-Tato M.P., Estevinho L., Seijas J.A., Iglesias A. (2012). Organic bee pollen: Botanical origin, nutritional value, bioactive compounds, antioxidant activity and microbiological quality. Molecules.

[68] Conte G., Benelli G., Serra A., Signorini F., Bientinesi M., Nicolella C., Mele M., Canale A. (2017). Lipid Characterization of Chestnut and Willow Honeybee-Collected Pollen: Impact of Freeze-Drying and Microwave-Assisted Drying. J. Food Compos. Anal..

[69] GardanaDomenici V., Gabriele M., Parri E., Felicioli A., Sagona S., Pozzo L., Pucci L. (2015). Phytochemical composition and antioxidant activity of Tuscan bee pollen of different botanic origins. Ital. J. Food Sci..

[70] Taha E.-K.A., Al-Kahtani S., Taha R. (2019). Protein Content and Amino Acids Composition of Bee-Pollens from Major Floral Sources in Al-Ahsa, Eastern Saudi Arabia. Saudi J. Biol. Sci..

[71] Bryś M.S., Strachecka A. (2024). The Key Role of Amino Acids in Pollen Quality and Honey Bee Physiology—A Review. Molecules.

[72] de Arruda V.A.S., Pereira A.A.S., de Freitas A.S., Barth O.M., de Almeida-Muradian L.B. (2013). Dried Bee Pollen: B Complex Vitamins, Physicochemical and Botanical Composition. J. Food Compos. Anal..

[73] Human H., Nicolson S.W. (2006). Nutritional Content of Fresh, Bee-Collected and Stored Pollen of *Aloe Greatheadii* Var. *Davyana* (Asphodelaceae). Phytochemistry.

[74] Song Y., Zhang J., Luo Z., Wu L., Cai Z., Zhong X., Zeng X., Cao T., Chen H., Xu S. (2024). Association between Dietary Branched-Chain Amino Acids and Multiple Chronic Conditions among Older Adults in Chinese Communities. Nutr. Metab..

[75] Çobanoğlu D.N. (2024). Medicinal Bee Pollen: Composition and Therapeutic Properties. Plants as Medicine and Aromatics.

[76] Aylanc V., Falcão S.I., Vilas-Boas M. (2023). Bee Pollen and Bee Bread Nutritional Potential: Chemical Composition and Macronutrient Digestibility under in Vitro Gastrointestinal System. Food Chem..

[77] Li Q.-Q., Wang K., Marcucci M.C., Sawaya A.C.H.F., Hu L., Xue X.-F., Wu L.-M., Hu F.-L. (2018). Nutrient-Rich Bee Pollen: A Treasure Trove of Active Natural Metabolites. J. Funct. Foods.

[78] Hänsch R., Mendel R.R. (2009). Physiological Functions of Mineral Micronutrients (Cu, Zn, Mn, Fe, Ni, Mo, B, Cl). Curr. Opin. Plant Biol..

[79] Liolios V., Tananaki C., Papaioannou A., Kanelis D., Rodopoulou M.-A., Argena N. (2019). Mineral Content in Monofloral Bee Pollen: Investigation of the Effect of the Botanical and Geographical Origin. Food Meas..

[80] Bakour M., Fernandes Â., Barros L., Sokovic M., Ferreira I.C. (2019). Bee Bread as a Functional Product: Chemical Composition and Bioactive Properties. LWT.

[81] Valverde S., Tapia J.A., Pérez-Sanz A., González-Porto A.V., Higes M., Lucena J.J., Martín-Hernández R., Bernal J. (2023). Mineral Composition of Bee Pollen and Its Relationship with Botanical Origin and Harvesting Period. J. Food Compos. Anal..

[82] Sattler J.A.G., DE-MELO A.A.M., do Nascimento K.S., de Melo I.L.P., Mancini-Filho J., Sattler A., de Almeida-Muradian L.B. (2016). Essential Minerals and Inorganic Contaminants (Barium, Cadmium, Lithium, Lead and Vanadium) in Dried Bee Pollen Produced in Rio Grande Do Sul State, Brazil. Food Sci. Technol..

[83] Altunatmaz S.S., Tarhan D., Aksu F., Barutçu U.B., Or M.E. (2017). Mineral Element and Heavy Metal (Cadmium, Lead and Arsenic) Levels of Bee Pollen in Turkey. Food Sci. Technol..

[84] Krejčí P., Žingor Z., Balarynová J., Čevelová A., Tesárek M., Smỳkal P., Bednář P. (2025). Modern Comprehensive Metabolomic Profiling of Pollen Using Various Analytical Techniques. Molecules.

