# Supplementary material for: Characterization of Multifloral Bee Pollen Collected from Geographically and Botanically Distinct Regions in Tunisia
Source: Foods. 2025 Nov 21;14(23):3986. doi: 10.3390/foods14233986 (PMC12692326; doi:10.3390/foods14233986)
Supplement: Supplementary file 1 [file foods-14-03986-s001.zip › foods-3955271-supplementary.pdf]

SUPPLEMENTARY DATA

Table S1. Geographical and bioclimatic characteristics of the seven bee pollen collection sites in Tunisia

Samples Code	Collection Site	Latitude (°N)	Longitude (°E)	Altitude (m)	Bioclimatic Zone	Notes
**A-04**	Bizerte-Aousja	37.28	9.87	15	Humid coastal	Northern Tunisia
**G-03**	Nabeul-Ghardiai	36.45	10.73	25	Sub-humid coastal	Near Mediterranean coast
**K-04**	Kairouan	35.68	10.10	65	Semi-arid central	Plains region
**K-09**	Kairouan-Weslatia	35.65	10.05	70	Semi-arid central	Slightly inland
**M-05**	Bizerte-Mateur	37.05	9.72	20	Humid coastal	Coastal plain
**O-04**	Bizerte-Om Heni	37.05	9.70	18	Humid coastal	Close to Mateur
**T-03**	Tozeur	33.92	8.13	55	Arid southern desert	Oasis region

Table S2. Fatty acids identified by GC-MS on bee pollen samples from Tunisia

No	t_R (min)	C:D	Fatty acids
**1**	4.32	C6:0	Caproic acid
**2**	5.08	C8:0	Octanoic acid
**3**	6.18	C10:0	Capric acid
**4**	7.16	C12:0	Lauric acid
**5**	8.22	C14:0	Myristic acid
**6**	9.70	C16:0	Palmitic acid
**7**	9.96	C16:1	Palmitoleic acid
**8**	11.74	C18:0	Stearic acid
**9**	12.04	C18:1n9c	Oleic acid
**10**	12.65	C18:2n6c	Linoleic acid
**11**	13.56	C18:3n3	Linolenic acid
**12**	14.51	C20:0	Arachidic acid
**13**	14.88	C20:1	Gondoic acid
**14**	17.94	C22:0	Behenic acid
**15**	18.56	C22:1	Erucic acid
**16**	24.13	C24:0	Lignoceric acid

Table S3. Amino Acids identified by HPLC-FLD on bee pollen samples from Tunisia

No	Amino Acid	Formula	Molecular Weight (g/mol)	t_R (min)
**1**	Aspartic acid	$C_4H_7NO_4$	133.10	2.24
**2**	Glutamic acid	$C_5H_9NO_4$	147.13	2.59
**3**	Serine	$C_3H_7NO_3$	105.09	3.38
**4**	Glycine	$C_2H_5NO_2$	75.07	3.84
**5**	Threonine	$C_4H_9NO_3$	119.12	4.21
**6**	Alanine	$C_3H_7NO_2$	89.09	4.68
**7**	Arginine	$C_6H_{14}N_4O_2$	174.20	5.07
**8**	Tyrosine	$C_9H_{11}NO_3$	181.19	5.56
**9**	Valine	$C_5H_{11}NO_2$	117.15	6.32
**10**	Histidine	$C_6H_9N_3O_2$	155.15	7.22
**11**	Phenylalanine	$C_9H_{11}NO_2$	165.19	7.54
**12**	Isoleucine	$C_6H_{13}NO_2$	131.18	8.23
**13**	Leucine	$C_6H_{13}NO_2$	131.18	8.63
**14**	Lysine	$C_6H_{14}N_2O_2$	146.19	9.11

**15**	Proline	$C_5H_9NO_2$	115.13	10.51
**16**	Tryptophan	$C_{11}H_{12}N_2O_2$	204.23	12.44
**17**	Cysteine	$C_3H_7NO_2S$	121.16	13.88
